# Indications for zygomatic implants: a systematic review

**DOI:** 10.1186/s40729-023-00480-4

**Published:** 2023-07-01

**Authors:** Waldemar D. Polido, Agustin Machado-Fernandez, Wei-Shao Lin, Tara Aghaloo

**Affiliations:** 1 Department of Oral and Maxillofacial Surgery, Indiana University School of Dentistry, Indianapolis, USA; 2 Department of Oral and Maxillofacial Surgery, PUCRS School of Dentistry, Porto Alegre, RS Brazil; 3 Department of Prosthodontics, Indiana University School of Dentistry, Indianapolis, USA; 4 Section of Oral and Maxillofacial Surgery, UCLA School of Dentistry, Los Angeles, CA USA

**Keywords:** Zygomatic implants, Zygoma implants, Atrophic maxillae, Zygoma

## Abstract

**Purpose:**

The purpose of this systematic review was to assess the evidence regarding the indications for placement of zygomatic implants to rehabilitate edentulous maxillae.

**Material and methods:**

A focused question using the PIO format was developed, questioning “in patients in need of an implant-supported rehabilitation of the edentulous maxillae, what are the indications for the use of zygomatic implants’’. The primary information analyzed and collected was a clear description of the indication for the use of zygomatic implants.

**Results:**

A total of 1266 records were identified through database searching. The full-text review was conducted for 117 papers, and 10 were selected to be included in this review. Zygomatic implant indications were extreme bone atrophy or deficiency secondary to different factors. The quad zygoma concept (two zygomatic implants bilaterally placed and splinted) was applied to 107 patients, the classic zygoma concept (one zygomatic implant bilaterally placed and splinted to standard anterior implants) was used in 88 patients, and the unilateral concept (one zygomatic implant on one side, splinted with one or more conventional implants) was employed in 14 patients.

**Conclusions:**

The main indication for the use of zygomatic implants was considered extreme maxillary bone atrophy, resulting from many factors. The clear definition of what was considered “extreme bone atrophy” is not uniquely defined in each paper. Further studies are needed to develop clear indications for zygomatic implants.

**Graphic Abstract:**

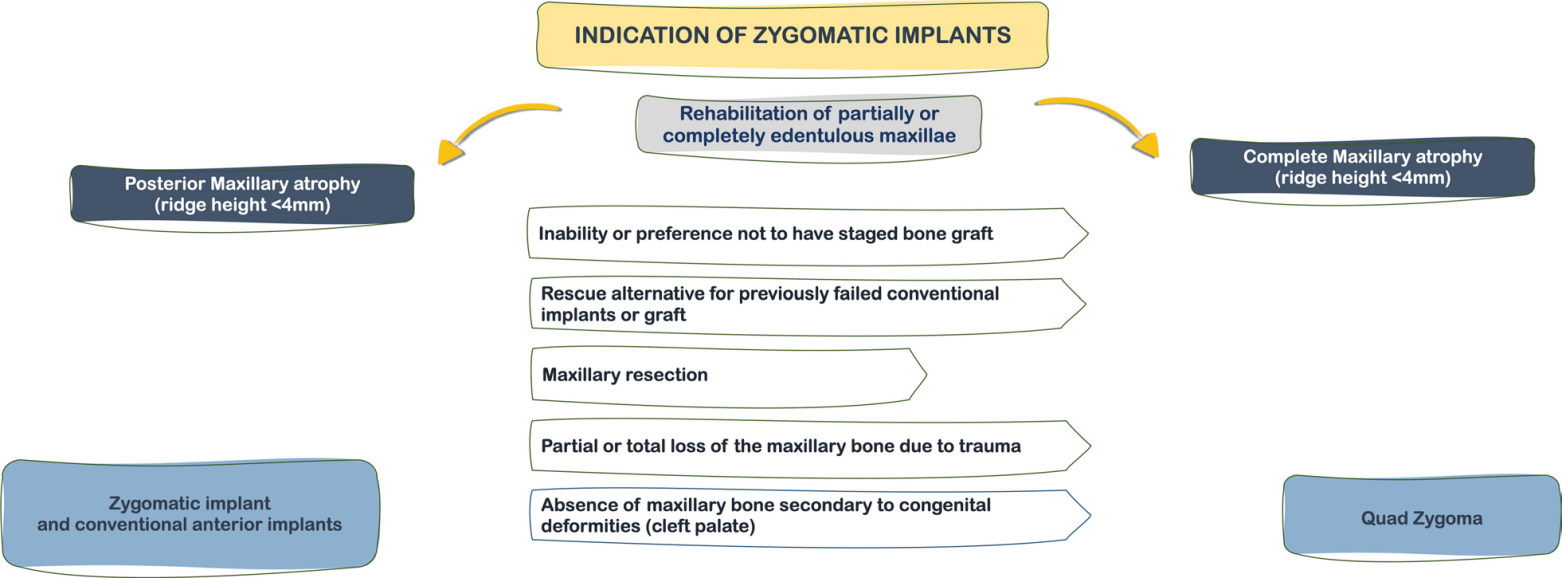

## Introduction

Maxillary edentulism is a growing condition worldwide. According to the World Health Organization (WHO), losing teeth is generally the endpoint of a lifelong history of oral disease, mainly advanced dental caries, and severe periodontal disease. But it can also occur from trauma, pathology, infection, and other causes. The estimated global average prevalence of complete tooth loss is approximately 7% among people aged 20 years or older. For people aged 60 years or older, a much higher global prevalence of 23% has been estimated. Losing teeth can be psychologically traumatic, socially damaging and functionally limiting [1].

The American College of Prosthodontists estimates that in the geriatric population the ratio of edentulous to dentate individuals is 2 to 1. Approximately 23 million are completely edentulous and about 12 million people are edentulous in one arch. Adverse consequences of edentulism are restricted possibility of food consumption, due to the inability to chew, which may cause include significant nutritional changes, obesity, diabetes, coronary artery disease, and some forms of cancer [2].

After tooth loss, resorption of the alveolar bone in the maxilla occurs in a posterior/superior and lateral-to-medial direction. Pneumatization of the sinuses, added to alveolar bone resorption, may lead to a limited vertical and horizontal bone volume in the posterior region. Lack of adequate anterior alveolar bone resorption may reduce the possibility of utilizing conventional implants. The prolonged use of complete dentures may increase the severity of maxillary atrophy [3, 4].

Several bone augmentation procedures have been developed to address this problem, such as sinus floor elevation procedures, onlay grafts and interpositional osteotomies [5–7]. Although these ancillary procedures have been researched and improved for many years, success rates are variable. Even though these procedures are successful, there is an increased risk of higher morbidity, longer treatment times, extended periods without a prosthesis, and a high dependence on the surgeons’ surgical preference and expertise [8, 9].

Graftless and graft-less alternatives have been discussed to reduce risks, morbidity, and treatment time, leading to more predictable outcomes [10, 11]. These types of treatment are often preferred by patients, considering that they may reduce total treatment time and have less morbidity than staged bone augmentation procedures [12–15].

Zygomatic implants were developed and introduced by Prof. P-I Brånemark and were originally designed to obtain stable prosthesis retention in edentulous patients with extreme maxillary atrophy or oncologic patients that had partial or complete maxillary resection, who were not suitable for conventional dental implant placement. The original zygomatic Brånemark protocol included one implant on each zygoma, traversing the sinus, and splinted to 2 to 4 conventional implants in the anterior region [16]. The zygomatic implants offer anchorage for a fixed bridge using less invasive surgery compared with bone augmentation procedures [17–19]. Since then, many modifications to zygomatic implant designs, surgical approaches and loading protocols have been documented in the literature [20–28].

Over the past 20 years, indications for zygomatic implants have evolved to include severe posterior maxillary resorption with insufficient bone for conventional implant placement, with or without previously failed implant or bone graft treatment. Other indications described in the literature include patients with maxillary deficiency secondary to cleft palate, failed conventional implant therapy, unsuccessful bone grafting or refusal to undergo bone grafting. Patients that underwent complete or partial maxillectomy secondary to benign or malignant tumor resections are still one of the main reported uses for zygomatic implants, assisting in supporting obturators and/or removable prostheses [18, 29–31]. In cases without adequate anterior maxillary bone, the quad zygomatic implant concept was introduced, where two zygomatic implants are bilaterally placed (two on each side), and splinted, providing acceptable antero-posterior distribution and adequate biomechanics [29, 32].

Even though the insertion of zygomatic implants still is a very complex procedure with significant surgical risks and potential complications, its use has grown exponentially, having documented high survival rates [33–36]. In a recent position paper, the American College of Prosthodontists affirms that zygomatic implants in various clinical scenarios with multiple configurations enable the dental team to restore quality of life and provide an expedited and predictable option [2, 37, 38].

What is unclear in the literature is when zygomatic implants should be utilized instead of traditional bone grafting procedures or other graftless or graft-less alternatives. Many papers cite “severe maxillary atrophy” or “atrophic maxilla” without defining the degree of bone resorption or available bone [39–46]. Moreover, there have been many advances with conventional implants, where improved implant surfaces, materials, and strong evidence behind reduced diameter and short implants may allow for its  expanded use in atrophic situations [47, 48]. However, the possibility of shortened treatment time, including immediate loading, engagement of stable cortical bone in the zygoma, and the lack of need for grafting, has influenced the decision to utilize zygomatic implants to rehabilitate edentulous atrophic maxillae with an implant-supported prosthesis [14, 31, 49, 50].

Therefore, the purpose of this systematic review is to address the question “In patients in need of an implant-supported rehabilitation of the edentulous maxillae, what are the indications for the use of zygomatic implants?”

## Materials and methods

The current systematic review was reported following the Preferred Reporting Items for Systematic Reviews and Meta-analysis (PRISMA) statement. The PRISMA 2020 [51] provides updated reporting guidance for systematic reviews that reflects advances in methods to identify, select, appraise, and synthesize studies.

### PIO focused question

Since we were not comparing the indications with other procedures, a focused question was formulated and approved by all authors, using the PIO format, questioning if “In patients in need of a maxillary implant-supported rehabilitation, what are the indications for the use of zygomatic implants?”.

Population was defined as maxillary completely or partially edentulous patients (or those who are to become), that had implant-supported prostheses (fixed or removable); intervention was defined as zygomatic implants (unilateral, bilateral) supporting fixed or removable maxillary prostheses; outcomes assessed were successful rehabilitations with a fixed or removable implant-supported prostheses, involving zygomatic implants.

### Data source and eligibility criteria

A systematic search of the PubMed, EMBASE and Google Scholar databases was performed, being last updated on October 31, 2022. All databases were searched from inception to October 31, 2022. Only articles written in the English language were considered.

The search strategy employed the following medical subject heading (Mesh) terms for Pubmed and Emtree terms and their synonyms for Embase that were found related to the PIO question in the databases; P (Jaw, Edentulous maxilla), I (Full mouth rehabilitation), O (successful rehabilitations with fixed or removable implant-supported prostheses, involving zygomatic implants). The following words were used as free words due to not being Mesh terms or Emtree terms (Zygomatic implants, Quad Zygoma, Conventional implants, Graft). The final search strategy utilized is described below:

(Maxillas OR “Maxillary Bone” OR “Bone, Maxillary” OR “Bones, Maxillary” OR “Maxillary Bones” OR Maxillae OR “edentulous maxilla” OR “Jaw, Edentulous” OR maxilla OR “Edentulous Jaw” OR “Edentulous Jaws” OR “Jaws, Edentulous” OR “jaw, upper” OR maxillary OR “maxillary area” OR “upper jaw”) AND (“Zygomatic implants” OR “Zygomatic implant” OR “Quad zygoma” OR “full arch dental reconstruction” OR “full arch reconstruction” OR “full arch rehabilitation” OR “full arch restoration” OR “full mouth reconstruction” OR “full mouth restoration” OR “mouth rehabilitation” OR “full arch prosthesis” OR “full mouth rehabilitation”) AND (“Conventional implants” OR Graft OR “full mouth” OR “rehabilitation Implant, Dental” OR “Implants, Dental” OR “Dental Implant” OR “Dental Prostheses, Surgical” OR “Dental Prosthesis, Surgical” OR “Surgical Dental Prostheses” OR “Surgical Dental Prosthesis” OR “Prostheses, Surgical Dental” OR “Prosthesis, Surgical Dental” OR “tooth implant” OR “implant, teeth” OR “implant, tooth” OR “implants, teeth” OR “implants, tooth” OR “Bone graft” OR “Autograft, bone” OR “autograft, spongy bone” OR “autologous bone” OR graft OR “bone autograft” OR “bone flap” OR “bone flaps” OR “bone grafts” OR “bone transplant” OR “Bone Ceramic” OR “compact bone” OR autograft OR “free bone graft” OR “graft, bone” OR “osseous flap” OR “osseous flaps” OR “osseous graft” OR “osseous grafts” OR “osteoarticular graft” OR “rib autograft” OR “spongy bone” OR autograft).

For the Google Scholar search, the same strategy was used.

The reference lists of all articles retrieved through the main search and grey literature strategy were manually searched for additional relevant papers.

### Inclusion and exclusion criteria

Inclusion criteria were considered as: utilized zygomatic implants to support a dental prosthesis; included at least 10 patients with a minimum follow-up period of 12 months; clearly stated the indications for the use of zygomatic implants.

Studies comparing zygomatic implants to any other implant therapy including grafted sites were considered, as well as oncologic rehabilitation using zygomatic implants. Randomized clinical trials, prospective and retrospective studies, and case series were considered if the selection criteria were met.

Animal and in vitro studies were not considered. The exclusion criteria also applied to papers where there was no clear definition for the indication for use of zygomatic implants.

### Study selection

Systematic database searches were performed as described by one author (AMF). Duplicates were removed and the remaining studies were independently screened and selected by two authors (AMF and WSL). A standardized form was created using the inclusion and exclusion criteria to facilitate and maintain consistency of eligibility. After an analysis of titles and abstracts, the articles were evaluated following the eligibility criteria.

Kappa statistic of interrater reliability was performed. Cohen’s *k* was run to determine the agreement between the two authors during paper selection. For title and abstract reviews, there was good agreement between the two authors, *k* = 0.899 (95% agreement rate, confidence interval 0.888 until 0.909). According to Landis and Koch [52], this is considered an “almost perfect” observer agreement. All papers that met the inclusion criteria and agreed by the authors were selected for full-text reading.

During the full-text review, a third author (WDP) decided whether to include or exclude an article.

Studies that did not meet the inclusion criteria were excluded, and the reason for exclusion was recorded. When the indication referred only to severe maxillary atrophy and/or the use only of some classification (Cawood and Howell, Bedrossian, Lekholm and Zarb, Misch, Brown), but the study did not cite any other indication, it was excluded.

### Data extraction

Data were extracted from each of the identified eligible studies, and tabulated including:author, year of publication, type of study, number of patients, number of zygomatic implants placed, distribution of zygomatic implants (unilateral, bilateral or quad), additional conventional implants placed, follow-up time, loading protocol and the description of the indication (extreme bone resorption; avoid bone graft; maxillectomy secondary to pathology; cleft palate; trauma; previous unsuccessful treatment).

The primary information analyzed and collected was a clear description of the zygomatic implant indication. Secondary outcomes were the distribution of implants and loading protocols.

### Risk of bias

The risk of bias was assessed based on the type of study available. Only one study was an RCT, and it was assessed utilizing the Cochrane RoB 2 tool [53]. The remaining non-RCT papers were assessed using the ROBINS-I (Risk of Bias in Non-randomized Studies—of Intervention) [54]. For the bias analysis of the RCT study, it was considered confounding factors, selection of participants to the study, classification of interventions, deviations from the intended intervention, missing data, measurement of outcomes, and selection of reported results. For non-RCT studies, the criteria considered sample selection (selection bias), allocation concealment (selection bias), blinding of participants and personnel (performance bias), blinding of outcomes assessment (detection bias), incomplete outcome data (attrition bias), selective reporting (reporting bias), other bias, and overall bias.

Studies were classified as having a low risk of bias if all items were present, a medium risk of bias if one or two items were missing, and a high risk of bias if three or more items were missing.

The reviewers (AMF and WDP) ranked each study independently and resolved disagreements by reciprocal consulting.

## Results

**Fig. 1:**
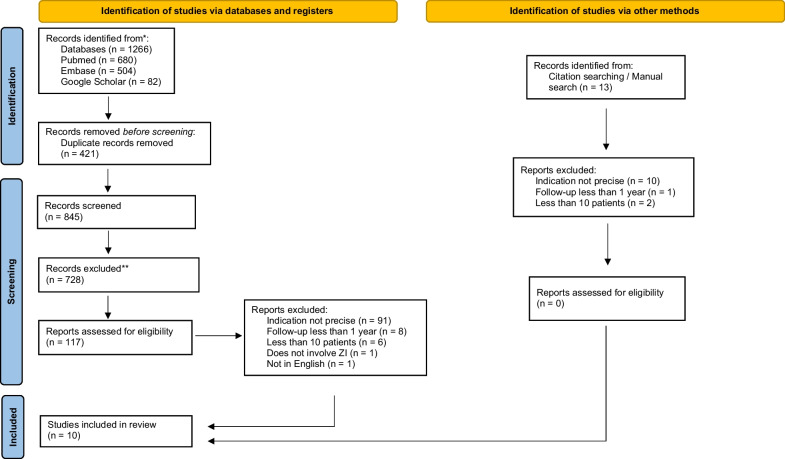
PRISMA flow diagram

A total of 1266 records were identified through database searching, 680 on PubMed, 504 on Embase and 82 on Google Scholar. Duplicates (*n* = 421) were removed and a total of 845 documents had titles and abstracts screened by two authors (AMF and WSL). Of those, 728 records were excluded, and full-text review was requested for 117 papers. From those, 10 were selected to be included in this review. The PRISMA flow diagram is shown in Fig. 1.

Additional 13 records were identified by manual and citation searches. All 13 reports were excluded. Ten that did not specify the precise indication, 2 had less than 10 patients and 1 had a follow-up for less than 12 months.

The main reasons for exclusion were follow-up of fewer than 12 months, less than 10 patients, not involving a zygomatic implant, and full text not in English. Most of the excluded papers failed to report a clearly described indication for the use of zygomatic implants, but rather cited potential advantages of zygomatic implants such as immediate loading, as an indication.

The selected papers included the use of 622 zygomatic implants in 209 patients, with a median follow-up of 28.5 months (range 12–162 months). The mean reported survival rate for zygomatic implants was 97% (89–100%). All data extracted are listed in Table 1.

**Table 1:** Selected papers with all data extracted

Author/year	Study design	Sample size	Number of ZI	Number of CI	Follow-up (months)	Mean age	ZI survival rate (%)	Classification	Indications							Zygoma concept		Loading protocol	
									Extreme bone resorption	Avoid graft	Medical considerations	Previous unsuccessful treatment	Cleft palate	Cancer	Unilateral	Bilateral	Quad zygoma	Conventional	Immediate
Becktor et al*.*, 2005	Retrospective	*N* = (16) *n* = (15)	30	74	46.4 (9–69)		94.3	Cawood and Howell		10	1	4				15		16	
Ahlgren et al*.*, 2006	Retrospective	13	25	28	35 (11–49)	59 (49–73)	100	No	5		1	7	1		1	12		13	
Landes et al*.*, 2009	Retrospective	15	36	24	64.2 (13–102)	58 (24–79)	89	No			15		3	10	7	7	1	15	
Stiévenart et al*.*, 2010	Retrospective	*N* = (20) *n* = (19)	76		12	56 (35–75)	96	Lekholm and Zarb		19							19	10	9
Muñoz et al*.*, 2017	Retrospective	10	40		24	57.7 (41–78)	100	No	7			3					10		10
Atalay et al*.*, 2017	Retrospective	16	32	38	28 (6–96)	53 (23–68)	93.7	Cawood and Howell	10		6			5				16	
Davó et al*.*, 2018	RCT	35	128	13	12	58 (43–74)	96.9	No	35								35		35
Blanc et al*.*, 2020	Retrospective	25	76	64	18.6 (12–26)		100	Cawood and Howell	19		2	4	1	1	4	7	14		25
D'Agostino et al*.*, 2021	Retrospective	42	116	70	60 (12–162)	54 (24–76)	97.4	Cawood and Howell	31		3	8	2			26	16	35	6
Laventure et al*.*, 2022	Retrospective	*N* = (22) *n* = (19)	63	27	36.2 (13–103)	63 (46–80)	97.3	Cawood and Howell	11			8				7	12		19
Total		209	622	338	28.5 (12–162)	57.2 (24–80)	97 (89–100)		118	29	5	34	7	16	12	74	107	105	104

*ZI* zygomatic implants, *CI* conventional implants, *N* total sample, *n* remaining patients after exclusion of any patients

Zygomatic implant indications were extreme bone atrophy or deficiency [n=118], unsuccessful previous treatments with grafts and/or implants [n=34], avoidance of staged bone graft procedures [n=29] and medical considerations that may complicate traditional bone grafting procedures, such as benign cysts, amelogenesis imperfecta and trauma [n=5].

The use of zygomatic implants was also indicated in cases associated with benign or malignant maxillary resections [n=16]. Those included resection secondary to osteosarcoma [n=1], squamous cell carcinoma [n=11], adenoid cystic carcinoma [n=1], mixed salivary carcinoma [n=2]. One paper did not report the type of pathology associated with the resection. Zygomatic implants to rehabilitate maxillary defects secondary to cleft palate were reported in 7 cases.

Five studies classified the degree of maxillary atrophy using the Cawood and Howell classification, one used the Lekholm and Zarb classification and four did not use a classification but listed actual measurements and anatomic descriptions of the treated patients.

The quad zygoma concept (four zygomatic implants, two on each side) was applied to 107 patients, the classic zygoma concept (bilateral, with one on each side splinted to conventional anterior implants) was used in 88 patients, and the unilateral concept (one zygomatic implant on one side, splinted with one or more conventional implants) was employed in 14 patients.

Immediate loading was employed in 104 patients and conventional loading in 105 patients.

### Risk of bias of selected studies

**Fig. 2:**
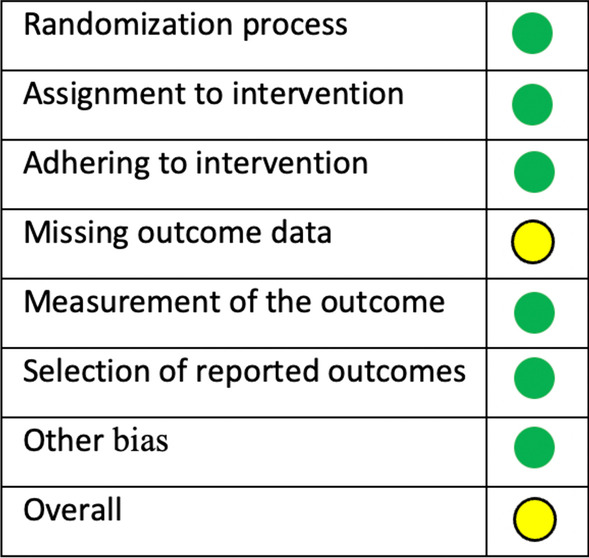
Risk of bias RCT (RoB 2)

**Fig. 3:**
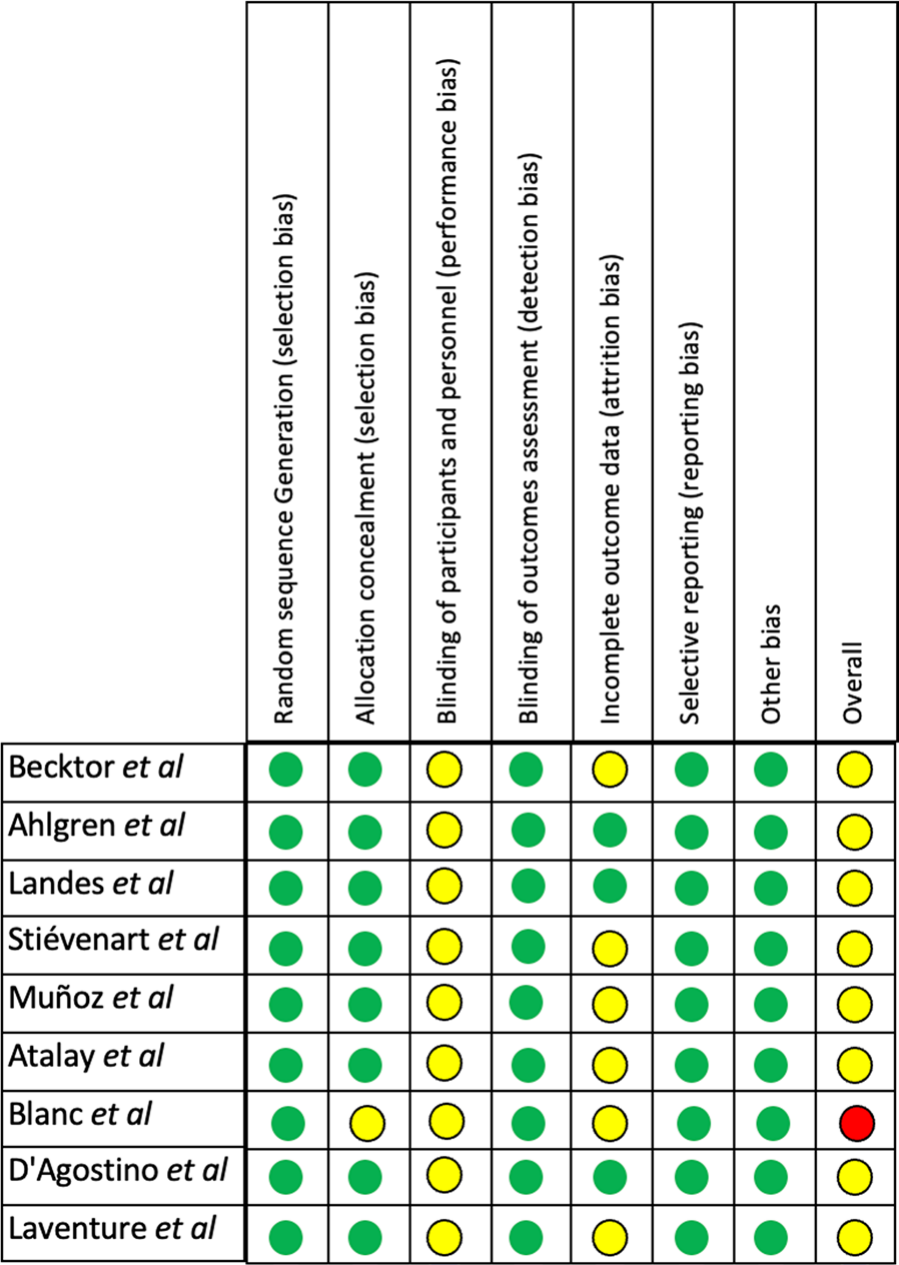
Risk of bias of non-RCT (ROBINS-I)

The risk of bias for the included papers is shown in Figs. 2 and 3. The risk of bias for the RCT included [14] was assessed utilizing the Cochrane RoB 2 tool [53], and the result was moderate (Fig. 2). The remaining non-RCT papers were assessed using the ROBINS-I tool [54]. Eight papers were classified to have a moderate risk of bias, and one [13] was considered with a high risk of bias (Fig. 3).

## Discussion

Zygomatic implants are considered a graftless solution to rehabilitate atrophic maxillae using a fixed or removable implant-supported prosthesis [26, 55]. To avoid extensive bone graft procedures, the concept of bone anchorage for a prosthesis is applied, instead of reconstructing the alveolar bone, creating conditions for ideal numbers, dimensions, and distribution of implants, while improving the final restoration.

Brånemark et al*.* reported that “the new zygoma fixture was a direct response to the acknowledged need for improvements in onlay grafting procedures, particularly for improved stability of fixtures and to minimize the need for further surgery”. At that time, the grafting alternatives for severely atrophic cases were mostly autogenous onlay and inlay grafts [16].

The original zygomatic Brånemark protocol included one implant in each zygoma traversing the sinus combined with two to four anterior conventional implants [16]. Since then, many modifications to zygomatic implant designs, surgical approaches, and loading protocols have been documented in the literature [20–28]. However, the original indication for zygomatic implants (maxillary defects secondary to maxillectomies) remains one of the main indications.

Maxillary ablative defects secondary to resection to treat benign or malignant tumors are listed as indications for zygomatic implants, to support maxillary obturators. In these major defects, grafting alternatives are complex and less predictable, and even if considered successful, they may not allow for the use of conventional implants. Hence, zygomatic implants may be the only remaining alternative to assist in maxillofacial prosthodontics rehabilitation. The same concept may apply to cleft patients that present with partial loss of the maxillary bone, where grafting alternatives may not be achievable. The team involved in the rehabilitation of complex defects may consider the zygomatic implant as a less complex alternative to support maxillofacial prosthodontics.

Indications for zygomatic implants in conventional edentulous patients are presented from different points of view in a variety of papers. The most common indication is “severe atrophy”. However, the published indications for zygomatic implants must be scrutinized, when compared to conventional treatment alternatives. Moreover, indications must be distinguished from advantages that arise from a successful treatment using zygomatic implants, such as patient and/or surgeon preference, avoidance of a grafting procedure, or the possibility of immediate loading. The use of an immediate loading protocol may be a possibility when using zygomatic implants, and advantageous for the patients. However, it is not a clear indication of it, as the loading protocol is dependable on the surgical and restorative team’s expertise and intra-operative findings and may not be always employed.

It is important to understand that when zygomatic implants were originally introduced, the implantology community had mainly autogenous bone grafting techniques as an alternative, followed by longer and regular diameter smooth surface implants. The development of bone graft substitutes, modern micro-rough surfaces, and the growing evidence behind reduced diameter, short and ultra-short implants may have changed what clinicians previously considered as a minimum available bone for conventional implant placement [47, 48]. In other words, patients with severe resorption requiring zygomatic implants in the past may be successfully treated today with non-autogenous bone substitutes with or without narrow diameter or short implants. The use of cone beam CT and imaging software also allows clinicians to perform a more accurate analysis of the alveolar and midface structures, including bone quality and volumetric measurements, and the possibility of virtual planning and guided surgery.

Out of the ten selected papers, five used the Cawood and Howell classification to define the degree of atrophy [6, 13, 56–58], four did not use any classification [14, 18, 59, 60], and one used the Lekholm and Zarb classification [29].

Thirty-five papers used a specific classification, but due to other missing information, not all were selected and included in the final review. Cawood and Howell was the most cited classification with 24 papers [31, 35, 49, 50, 61–80], followed by Bedrossian (5 papers) [7, 81–84], Lekholm and Zarb (2 papers) [28, 30], Misch (2 papers) [85, 86] and Brown (2 papers) [87, 88].

Published in 1988 and based on an analysis of 300 dry skulls, the Cawood and Howell classification describes the degree of atrophy according to alveolar bone remodeling, defining a class V or VI as remaining basal bone for both anterior and posterior regions of the maxilla and mandible. Measurements from the graphics of the original publication suggest that moderate and advanced resorption groups (Class V and VI) had a mean alveolar bone height of 1.09 mm (SD 1.45) in the anterior region and 6.46 mm (SD 2.54) in the posterior. The basal bone measurements suggest that basal bone height and width for both Classes were similar, with a mean of 10 mm for the anterior and 3 mm for the posterior regions [89].

Bedrossian et al*.* [90] described a systematic pretreatment evaluation method, looking at the presence or absence of a composite defect, visibility of the residual ridge crest, and the description of zones 1 (anterior), 2 (pre-molars) and 3 (posterior) to radiographically define presence or absence of bone in these 3 zones to define the best surgical approach. This protocol has been later refined and described in more detail [91]. According to this classification, zygomatic implant indications are defined according to the presence or absence of alveolar bone in zones 1, 2 and 3. This bi-dimensional zone classification is helpful to screen the availability of bone, but it does not give a clear definition of what is considered adequate bone to place a conventional implant. The use of two zygomatic implants splinted to at least two anterior implants is indicated when no bone is present in zone 3 and available in zones 1 or 2, and the use of four zygomatic implants is indicated when the bone is absent in zones 1, 2 and 3. The authors recognize that the Zones classification is helpful to screen for the presence of bone, but that a limitation of the protocol is the inability to assess the width of the existing bone, suggesting that the use of 3D imaging can precisely measure the width and height of the maxillofacial anatomy to help define the surgical alternatives and the outcome of the planned treatment [92].

Alveolar bone height was reported in 18 papers initially screened, but they were not included in the final selection due to other missing criteria. However, we looked at their reported alveolar bone height considered as an indication to place zygomatic implants (Table 2). Of those, 9 publications mention bone height being less than 4 mm [19, 21, 33, 93–98], four papers mention less than 3 mm [23, 34, 55, 99], 3 papers mention less than 5 mm [25, 100, 101], one paper mentions 2 mm or less [32] and one paper mentions less than 8 mm [102] (Table 2). The average alveolar bone height in the posterior maxilla reported in those papers was 4 mm, also the measurement reported in most of the papers (9 out of 18). However, these data show that there is no agreement on the minimum remaining alveolar bone to place conventional implants with or without additional grafting and indicate zygomatic implants. Neither the exact location where the bone height was measured nor any information about the width of the remaining alveolar ridge was in the paper. The 3D measurement of the remaining alveolar ridge is rarely presented in the publications.

**Table 2:** Articles that mentioned posterior alveolar ridge dimensions (in mm)

Author	Year	Sample size	Number of ZI placed	Indication per mm
Malevez et al.	2004	55	103	5
Aparicio et al.	2006	69	112	4
Bedrossian et al.	2006	14	28	3
Duarte et al.	2007	12	48	2
Aparicio et al.	2010	25	47	4
Aparicio et al.	2008	20	36	4
Migliorança et al.	2012	21	40	3
Davó et al.	2013	30	68	5
Aparicio et al.	2014	22	41	4
Aparicio et al.	2014	102	197	4
Yates et al.	2014	25	43	8
Espósito et al.	2017	20	80	4
Zhao et al.	2018	25	84	3
Balaji et al.	2020	11	19	4
Tao et al.	2020	23	72	3
Carvalho et al.	2021	31	55	4
Aparicio et al.	2021	122	488	5
Borgonovo et al.	2021	23	46	4

In severe atrophy, avoidance of extensive staged bone grafting and immediate loading are potential benefits of zygomatic implants, but not indications. This was a challenge in reviewing the literature, as benefits were often cited as indications.

Assuming that there is a biomechanical advantage of splinting implants placed to rehabilitate completely edentulous patients with an implant-supported prosthesis, and that the facial and lip support needs are the same irrespective of the type of implants placed, options such as distally tilted implants splinted to anterior implants, or short implants in the posterior maxilla in combination with reduced-diameter implants in the anterior maxilla, may provide the same support for the planned restorative solution as the use of zygomatic implants. Hence, in the assessment of maxillary atrophic bone to plan for implant placement, the use of short and reduced-diameter implants should be considered. However, their use is not well documented for full arch cases and for immediate loading protocols [47]. When the severe atrophy is presented only on the posterior maxilla, with relatively good bone height and width in the anterior maxilla, the placement of 4 implants, of which the posterior two are angulated distally, was well documented and allows the use of the immediacy concept [117].

The loading protocol is another point of discussion when defining indications for zygomatic implants. From our selected papers, 105 patients received conventional loading, and 104 had immediate loading. One study was excluded from this analysis because it did not present the distribution or the loading condition of implants [57]. A recent overview of systematic reviews [103] about zygomatic implants found that immediate loading was considered the primary treatment option because it provides function without having to wait for the conventional healing time when using delayed protocols. Bedrossian et al*.* [55] and Neugarten et al*.* [104] described a detailed protocol for immediate loading. However, they highlighted that this treatment should be reserved only for clinicians with experience in both surgical and restorative aspects.

The concept of immediacy is beneficial to patients, allowing them to achieve their desired outcome in a faster manner than using conventional loading protocols, which can add 4–6 additional months to the treatment, sometimes for a long period without an adequate interim restoration. Polido et al*.* [27] emphasized that immediate loading for full arch cases requires the utmost level of collaboration between the surgical and restorative teams, and this is certainly even more critical when using zygomatic implants. Although the zygomatic bone has usually adequate density and can allow for bicortical anchorage of the tip of the implant [105], the implant’s unique trajectory, and the frequent need to have the emergency directed towards the palatal region, may complicate or even contraindicate the application of immediate loading. Soft tissue aspects, swelling, and difficulty in properly seating the restoration and adjusting its occlusion may also influence the outcomes.

Immediate loading when using zygomatic implants is frequently reported with high survival rates [23, 31, 50, 70, 76, 101]. Our review shows that the immediate loading protocol was the loading protocol reported in recent publications, indicating a growing trend in this direction. However, the reports do not mention how many patients were scheduled for immediate loading but were unable to undergo it because of intraoperative or immediate postoperative factors. Therefore, all treatment options must be considered, and patients informed of possible treatment modifications if immediate loading is not possible. No articles reported on the 3D volume shape and/or density of the zygomatic bone itself. Although this is not a clear indicator of indication, it may play a role in surgical technique and the possibility of immediate loading.

The patient’s preference is also listed as an indication in a few reports [12, 57, 106]. These papers were excluded since they did not provide a clear criterion for the indication. However, when presented with treatment alternatives that differ in invasiveness, total treatment time and loading protocol, patients frequently prefer the procedure that has less morbidity and a reduced treatment time [12]. In a study evaluating patient satisfaction and implant survival rate in graftless alternatives, a mean patient satisfaction rate of 83% and a survival rate of 98% were obtained for zygomatic implants. In comparison, average patient satisfaction was 94% for tilted implants and 89% for short implants, with similar survival rates [12]. Zygomatic implants cannot be considered a minimally invasive procedure because they require larger flaps and bone exposure and involve important anatomic structures of the midface.

There is a recent growth in the utilization of zygomatic implants as a chosen alternative in comparison to simultaneous or staged grafting options, due to the possibility of a faster treatment time and immediate loading. However, zygomatic implant surgery and rehabilitation are considered one of the most complex procedures, requiring a higher expertise level from the surgical and restorative team [107]. Therefore, the indication must be very strictly evaluated in the routine treatment of edentulous patients and the surgical procedure remains reserved for experts. Most of the papers studied during the preparation of this manuscript emphasize the growing use of zygomatic implants, and the increased number of complications when performed by non-experienced surgeons. There is a need for a surgeon’s experience and expertise in maxillofacial surgical procedures in the midface, combined with an expertise in implant placement surgery.

Two recent reviews on the quality of systematic reviews and meta-analyses about zygomatic implants concluded that although this technique has been assessed and published for over 10 years, there is a limited number of systematic reviews about it, and they require a higher methodological rigor to provide more reliable results to professionals and patients [103, 108].

An adequate prosthetic plan and a detailed 3D imaging analysis are mandatory to assess all surgical alternatives and their associated risks and suggest an adequate treatment plan. Potential short- and long-term complications, treatment time, invasiveness, and cost are factors that need to be considered. The correct choice of approach and proper execution from the team are of paramount importance and have a major impact on the treatment outcomes.

Contra-indications generally include any general contra-indication to the surgical procedure and anesthesia, such as immunocompromised patients, pregnant patients, uncontrolled diabetes, acute sinusitis and drug or alcohol addiction [83]. Furthermore, radiation to the head and neck region with more than 70 Gy and medical treatment with bisphosphonates is also listed as general contra-indications. Reported local contra-indications are limited mouth opening (< 30 mm), acute maxillary sinusitis, chronic maxillary sinusitis with obstruction of the osteo-meatal complex, and any abnormality with the zygomatic bone [109]. Smokers and medical diseases that can be controlled before the procedure were considered relative contra-indications [38, 71, 110, 111].

## Conclusions

The literature is consistent in recommending zygomatic implants for the rehabilitation of partially or completely edentulous maxillae in unilateral, bilateral or quad zygoma concepts, where there is a moderate or severe atrophy in the posterior and/or anterior maxilla.

The main indications listed are: (a) patients without adequate alveolar bone for whom a staged bone graft would be indicated, but would not be desirable due to a medical compromise contra-indicating the grafting procedure; (b) rescue alternative for previously failed conventional implants or graft; (c) patient’s preference towards a graftless approach instead a staged grafting approach; (d) patients that had undergone maxillary resection secondary to pathology; (e) patients that had partial or total loss of the maxillary bone due to trauma; (f) patients with congenital deformities that led to the absence of maxillary bone, such as cleft palate.

However, the clear definition of what is considered a minimum amount of bone to allow for short and/or narrow implants or simultaneous implant placement and grafting procedures as alternatives for zygomatic implants is not clear from the studied papers. There is also a lack of studies reporting on full arch rehabilitation utilizing short and extra-short implants, combined or not with reduced-diameter implants, in a splinted fashion.

Therefore, we suggest that further studies can provide a better-defined indication for zygomatic implants by assessing the anatomy with three-dimensional imaging at each specific site, including the volume and density of the zygomatic bone, and by considering restorative needs, the patient’s condition and preferences, surgical alternatives, risks, and long-term outcomes.

The SAC classification in implant dentistry considers the treatment of extremely atrophic maxillae as a complex treatment, from both surgical and restorative aspects [118]. The final indication for the use of zygomatic implants must consider the type of restoration planned, the anatomy of the residual ridge and the zygomaticomaxillary region, the patient’s overall health and preferences, as well as the experience of the surgical and restorative teams.

## Data Availability

All data generated or analyzed during this study are included in this published article and its Additional files.

## References

[1] World Health Organization. 2022. https://www.who.int/news-room/fact-sheets/detail/oral-health. Accessed 9 Nov 2022.

[2] Tuminelli F, Balshi T. Zygomatic implants: position statement of the American College of Prosthodontics; 2016.

[3] Tallgren A (2003). The continuing reduction of the residual alveolar ridges in complete denture wearers: a mixed-longitudinal study covering 25 years. J Prosthet Dent.

[4] Branemark PI, Gröndahl K, Worthington P. The challenge of the severely resorbed maxilla. In: Darle C, editor. Osseointegration and autogenous onlay bone grafts: reconstruction of the edentulous atrophic maxilla. 2001. p. 2–6.

[5] Davó R (2009). Zygomatic implants placed with a two-stage procedure: a 5-year retrospective study. Eur J Oral Implantol.

[6] Laventure A, Lauwers L, Nicot R, Kyheng M, Ferri J, Raoul G (2022). Autogenous bone grafting with conventional implants vs zygomatic implants for atrophic maxillae: a retrospective study of the oral health-related quality of life. J Stomatol Oral Maxillofac Surg.

[7] Bedrossian E (2010). Rehabilitation of the edentulous maxilla with the zygoma concept: a 7-year prospective study. Int J Oral Maxillofac Implants.

[8] Chiapasco M, Casentini P, Zniboni M (2009). Bone augmentation procedures in implant dentistry. Int J Oral Maxillofac Implants.

[9] Aghaloo T, Misch C, Lin GH, Iacono V, Wang HL (2017). Bone augmentation of the edentulous maxilla for implant placement: a systematic review. Int J Oral Maxillofac Implants.

[10] Cooper LF, Thalji G, Al-Tarawneh S. Are nongrafting solutions viable for dental implant treatment in limited bone volume? 2020. www.compendiumlive.com. 32687381

[11] Misch C, Polido W (2019). A “graft less” approach for dental implant placement in posterior edentulous sites. Int J Periodontics Restor Dent.

[12] Pommer B, Watzek G, Doz Ass Bernhard Pommer P, Mailath-Pokorny G, Haas R, Busenlechner D (2014). Patients’ preferences towards minimally invasive treatment alternatives for implant rehabilitation of edentulous jaws. Eur J Oral Implantol.

[13] Blanc O, Shilo D, Weitman E, Capucha T, Rachmiel A (2020). Extramaxillary zygomatic implants: an alternative approach for the reconstruction of the atrophic maxilla. Ann Maxillofac Surg.

[14] Davó R, Felice P, Pistilli R, Barausse C, Marti-Pages C, Ferrer-Fuertes A (2018). Immediately loaded zygomatic implants vs conventional dental implants in augmented atrophic maxillae- 1-year post-loading results from a multicentre randomised controlled trial. Eur J Oral Implantol.

[15] Bedrossian E, Stumpel L, Beckely M, Indersano T (2002). The zygomatic implant: preliminary data on treatment of severely resorbed maxillae. A clinical report. Int J Oral Maxillofac Implants.

[16] Brånemark PI, Gröndahl K, Öhrnell LO, Nilsson P, Petrusen B, Svensson B (2004). Zygoma fixture in the management of advanced atrophy of the maxilla: technique and long-term results. Scand J Plast Reconstr Surg Hand Surg.

[17] Malevez C, Daelemans P, Adriaenssens P, Durdu F (2003). Use of zygomatic implants to deal with resorbed posterior maxillae. Periodontol 2000.

[18] Ahlgren F, Størksen K, Tornes K (2006). A study of 25 zygomatic dental implants with 11 to 49 months’ follow-up after loading. Case series. Int J Oral Maxillofac Implants.

[19] Aparicio C, Ouazzani W, Hatano N (2008). The use of zygomatic implants for prosthetic rehabilitation of the severely resorbed maxilla. Periodontol 2000.

[20] Stella JP, Warner MR (2000). Sinus slot technique for simplification and improved orientation of zygomaticus dental implants: a technical note. Int J Oral Maxillofac Implants.

[21] De Carvalho LF, De Carvalho LP, Sotto-Maior BS, Dias AL, Bezerra FJB, Bergamo ETP (2022). Rehabilitation of atrophic maxilla with immediate loading of extrasinus zygomatic implant. J Craniofacial Surg.

[22] Goker F, Grecchi F, Grecchi E, Bolzoni A, Del Fabbro M (2020). Insertion of zygomatic implants with a technical modification of the extrasinus protocol: a retrospective case series. Int J Oral Maxillofac Implants.

[23] Migliorança RM, Sotto-Maior BS, Senna PM, Francischone CE, Cury AADB (2012). Immediate occlusal loading of extrasinus zygomatic implants: a prospective cohort study with a follow-up period of 8 years. Int J Oral Maxillofac Surg.

[24] Aparicio C, Ouazzani W, Aparicio A, Fortes V, Muela R, Pascual A (2010). Extrasinus zygomatic implants: three year experience from a new surgical approach for patients with pronounced buccal concavities in the edentulous maxilla. Clin Implant Dent Relat Res.

[25] Aparicio C, Polido W, Chow J, David L, Davo R, De Moraes E (2021). Identification of the pathway and appropriate use of four zygomatic implants in the atrophic maxilla: a cross-sectional study. Int J Oral Maxillofac Implants.

[26] Aleksandrowicz P, Kusa-Podkańska M, Borgonovo A, Tomkiewicz W, Szczodry B, Kotuła L (2022). Finding better ways to perform graftless full rehabilitation of a compromised maxilla: new platform-switched zygomatic implants placed extra-sinus improve prosthetic restoration—a preliminary study of 25 cases and 85 implants. Int J Periodontics Restor Dent.

[27] Aparicio C, Polido WD, Chow J, Davó R, Al-Nawas B (2022). Round and flat zygomatic implants: effectiveness after a 1-year follow-up non-interventional study. Int J Implant Dent.

[28] Davó R, Malevez C, Rojas J, Rodríguez J, Regolf J (2008). Clinical outcome of 42 patients treated with 81 immediately loaded zygomatic implants: a 12-to 42-month retrospective study. Eur J Oral Implantol.

[29] Stiévenart M, Malevez C (2010). Rehabilitation of totally atrophied maxilla by means of four zygomatic implants and fixed prosthesis: a 6–40-month follow-up. Int J Oral Maxillofac Surg.

[30] Davó R, Pons O, Rojas J, Carpio E (2010). Immediate function of four zygomatic implants: a 1-year report of a prospective study. Eur J Oral Implantol.

[31] Davó R, Pons O (2015). 5-year outcome of cross-arch prostheses supported by four immediately loaded zygomatic implants: a prospective case series. Eur J Oral Implantol.

[32] Duarte LR, Filho HN, Francischone CE, Peredo LG, Brånemark PI (2007). The establishment of a protocol for the total rehabilitation of atrophic maxillae employing four zygomatic fixtures in an immediate loading system—a 30-month clinical and radiographic follow-up. Clin Implant Dent Relat Res.

[33] Aparicio C, Manresa C, Francisco K, Ouazzani W, Claros P, Potau JM (2014). The long-term use of zygomatic implants: a 10-year clinical and radiographic report. Clin Implant Dent Relat Res.

[34] Zhao K, Lian M, Fan S, Huang W, Wang F, Wu Y (2018). Long-term Schneiderian membrane thickness changes following zygomatic implant placement: a retrospective radiographic analysis using cone beam computed tomography. Clin Oral Implants Res.

[35] Bothur S, Kullendorff B, Olsson-Sandin G (2015). Asymptomatic chronic rhinosinusitis and osteitis in patients treated with multiple zygomatic implants: a long-term radiographic follow-up. Int J Oral Maxillofac Implants.

[36] Huang W, Wu Y, Zou D, Zhang Z, Zhang C, Sun J (2014). Long-term results for maxillary rehabilitation with dental implants after tumor resection. Clin Implant Dent Relat Res.

[37] Abd El Salam SE, El Khashab MA (2022). Zygomatic implants may improve quality of life and satisfaction in patients with atrophied maxilla. J Evid Based Dent Pract.

[38] Fernández-Ruiz JA, Sánchez-Siles M, Guerrero-Sánchez Y, Pato-Mourelo J, Camacho-Alonso F (2021). Evaluation of quality of life and satisfaction in patients with fixed prostheses on zygomatic implants compared with the all-on-four concept: a prospective randomized clinical study. Int J Environ Res Public Health.

[39] Farzad P, Andersson L, Gunnarsson S, Johansson B (2006). Rehabilitation of severely resorbed maxillae with zygomatic implants: an evaluation of implant stability, tissue conditions, and patients’ opinion before and after treatment. Int J Oral Maxillofac Implants.

[40] Peñarrocha M, García B, Martí E, Boronat A, Peñarrocha-Diago M (2007). Rehabilitation of severely atrophic maxillae with fixed implant-supported prostheses using zygomatic implants placed using the sinus slot technique: clinical report on a series of 21 patients. Int J Oral Maxillofac Implants.

[41] Peñarrocha M, Carrillo C, Boronat A, Martí E (2007). Level of satisfaction in patients with maxillary full-arch fixed prostheses: zygomatic versus conventional implants. Int J Oral Maxillofac Implants.

[42] Balshi SF, Glenn WJ, Thomas BJ (2009). A retrospective analysis of 110 zygomatic implants in a single-stage immediate loading protocol. Int J Oral Maxillofac Implants.

[43] Fernández H, Gómez-Delgado A, Trujillo-Saldarriaga S, Varón-Cardona D, Castro-Núñez J (2014). Zygomatic implants for the management of the severely atrophied maxilla: a retrospective analysis of 244 implants. J Oral Maxillofac Surg.

[44] Petrungaro PS, Gonzales S, Villegas C, Yousef J, Arango A (2020). A retrospective study of a multi-center case series of 452 zygomatic implants placed over 5 years for treatment of severe maxillary atrophy. Compend Contin Educ Dent.

[45] Nave P, Queralt A (2020). Zygomatic implants for the rehabilitation of atrophic maxillae: a retrospective study on survival rate and biologic complications of 206 implants with a minimum follow-up of 1 year. Int J Oral Maxillofac Implants.

[46] Vrielinck L, Blok J, Politis C (2022). Survival of conventional dental implants in the edentulous atrophic maxilla in combination with zygomatic implants: a 20-year retrospective study. Int J Implant Dent.

[47] Papaspyridakos P, De Souza A, Vazouras K, Gholami H, Pagni S, Weber HP (2018). Survival rates of short dental implants (≤6 mm) compared with implants longer than 6 mm in posterior jaw areas: a meta-analysis. Clin Oral Implants Res.

[48] Yu X, Xu R, Zhang Z, Yang Y, Deng F (2021). A meta-analysis indicating extra-short implants (≤ 6 mm) as an alternative to longer implants (≥ 8 mm) with bone augmentation. Sci Rep.

[49] Mozzati M, Gallesio G, Goker F, Tumedei M, Cesare P, Tedesco A (2021). Immediate oral rehabilitation with quad zygomatic implants: ultrasonic technique vs conventional drilling. J Oral Implantol.

[50] Maló P, Lopes A, Ferro A, Moss S, De M, Nobre A (2014). Five-year outcome of a retrospective cohort study on the rehabilitation of completely edentulous atrophic maxillae with immediately loaded zygomatic implants placed extra-maxillary. Eur J Oral Implantol.

[51] Page MJ, McKenzie JE, Bossuyt PM, Boutron I, Hoffmann TC, Mulrow CD, et al. The PRISMA 2020 statement: an updated guideline for reporting systematic reviews. BMJ. 2021;372. 10.1136/bmj.n71 PMC8005924 33782057

[52] Landis JR, Koch GG (1977). The measurement of observer agreement for categorical data. Biometrics.

[53] Revised Cochrane risk-of-bias tool for randomized trials (RoB 2). 2019. 10.1016/j.jclinepi.2020.06.015 32562833

[54] Sterne JA, Hernán MA, Reeves BC, Savović J, Berkman ND, Viswanathan M (2016). ROBINS-I: a tool for assessing risk of bias in non-randomised studies of interventions. BMJ.

[55] Bedrossian E, Rangert B, Eng M, Stumpel L, Indresano T (2006). Immediate function with the zygomatic implant: a graftless solution for the patient with mild to advanced atrophy of the maxilla. Int J Oral Maxillofac Implants.

[56] Becktor JP, Isaksson S, Abrahamsson P, Sennerby L (2005). Evaluation of 31 zygomatic implants and 74 regular dental implants used in 16 patients for prosthetic reconstruction of the atrophic maxilla with cross-arch fixed bridges. Clin Implant Dent Relat Res.

[57] Atalay B, Doǧanay Ö, Saraçoǧlu BK, Bultan Ö, Hafiz G (2017). Clinical evaluation of zygomatic implant-supported fixed and removable prosthesis. J Craniofacial Surg.

[58] D’Agostino A, Lombardo G, Favero V, Signoriello A, Bressan A, Lonardi F (2021). Complications related to zygomatic implants placement: a retrospective evaluation with 5 years follow-up. J Cranio-Maxillofac Surg.

[59] Alexandre Landes C, Paffrath C, Koehler C, Dung Thai V, Stübinger S, Sader R (2009). Zygoma implants for midfacial prosthetic rehabilitation using telescopes—9-year follow-up. Int J Prosthodont.

[60] Ruben M, Jose G, Alvaro D, Grace S (2017). Maxillary zygomatic hexagonal implant system (MZH system) for severe resorption: a new technique. Oral Maxillofac Surg.

[61] Maló P, de Araújo NM, Lopes A, Francischone C, Rigolizzo M (2012). Three-year outcome of a retrospective cohort study on the rehabilitation of completely edentulous atrophic maxillae with immediately loaded extra-maxillary zygomatic implants. Eur J Oral Implantol.

[62] Davó R, Pons O (2013). Prostheses supported by four immediately loaded zygomatic implants: a 3-year prospective study. Eur J Oral Implantol.

[63] Maló P, de Araújo NM, Lopes A, Ferro A, Moss S (2015). Extramaxillary surgical technique: clinical outcome of 352 patients rehabilitated with 747 zygomatic implants with a follow-up between 6 months and 7 years. Clin Implant Dent Relat Res.

[64] Mozzati M, Mortellaro C, Arata V, Gallesio G, Previgliano V (2015). Rehabilitation with 4 zygomatic implants with a new surgical protocol using ultrasonic technique. J Craniofacial Surg.

[65] Jensen OT, Adams MW, Butura C, Galindo DF (2015). Maxillary V-4- Four implant treatment for maxillary atrophy with dental implants fixed apically at the vomer-nasal crest, lateral pyriform rim, and zygoma for immediate function. Report on 44 patients followed from 1 to 3 years. J Prosthet Dent.

[66] Araújo PPT, Sousa SA, Diniz VBS, Gomes PP, da Silva JSP, Germano AR (2016). Evaluation of patients undergoing placement of zygomatic implants using sinus slot technique. Int J Implant Dent.

[67] D’Agostino A, Trevisiol L, Favero V, Pessina M, Procacci P, Nocini PF (2016). Are zygomatic implants associated with maxillary sinusitis?. J Oral Maxillofac Surg.

[68] Pellicer-Chover H, Cervera-Ballester J, Peñarrocha-Oltra D, Bagán L, Peñarrocha-Diago M, Peñarrocha-Diago M (2016). Influence of the prosthetic arm length (palatal position) of zygomatic implants upon patient satisfaction. Med Oral Patol Oral Cir Bucal.

[69] Hung KF, Ai QY, Fan SC, Wang F, Huang W, Wu YQ (2017). Measurement of the zygomatic region for the optimal placement of quad zygomatic implants. Clin Implant Dent Relat Res.

[70] Agliardi EL, Romeo D, Panigatti S, de Araújo NM, Maló P (2017). Immediate full-arch rehabilitation of the severely atrophic maxilla supported by zygomatic implants: a prospective clinical study with minimum follow-up of 6 years. Int J Oral Maxillofac Surg.

[71] Urgell JP, Revilla Gutiérrez V, Escoda CG (2008). Rehabilitation of atrophic maxilla: a review of 101 zygomatic implants. Med Oral Patol Oral Cir Bucal.

[72] D’Agostino A, Favero V, Nocini R, Venco J, Nocini PF, Trevisiol L (2019). Does middle meatal antrostomy prevent the onset of maxillary sinusitis after zygomatic implant placement?. J Oral Maxillofac Surg.

[73] Goker F, Grecchi E, Del Fabbro M, Grecchi F (2020). Clinical outcome of 302 zygomatic implants in 110 patients with a follow-up between 6 months and 7 years. Clin Implant Dent Relat Res.

[74] Peñarrocha-Diago M, Bernabeu-Mira JC, Fernández-Ruíz A, Aparicio C, Peñarrocha-Oltra D (2020). Bone regeneration and soft tissue enhancement around zygomatic implants: retrospective case series. Materials.

[75] Arcas-Sanabre AJ, Gutierrez-Santamaria J, López-López J, Ayuso-Montero R, Velasco-Ortega E (2020). Horizontal augmentation of the maxillary alveolar ridge to change the prosthetic profile: clinical and radiological results of a retrospective study. J Stomatol Oral Maxillofac Surg.

[76] Agliardi EL, Panigatti S, Romeo D, Sacchi L, Gherlone E (2021). Clinical outcomes and biological and mechanical complications of immediate fixed prostheses supported by zygomatic implants: a retrospective analysis from a prospective clinical study with up to 11 years of follow-up. Clin Implant Dent Relat Res.

[77] Wang F, Tao B, Shen Y, Li C, Huang W, Sun Y (2021). A single-arm clinical trial investigating the feasibility of the zygomatic implant quad approach for Cawood and Howell Class 4 edentulous maxilla: an option for immediate loading. Clin Implant Dent Relat Res.

[78] Lopes A, de Araújo Nobre M, Ferro A, Guedes CM, Almeida R, Nunes M (2021). Zygomatic implants placed in immediate function through extra-maxillary surgical technique and 45 to 60 degrees angulated abutments for full-arch rehabilitation of extremely atrophic maxillae: short-term outcome of a retrospective cohort. J Clin Med.

[79] Hernández-Alfaro F, Ragucci G, Valls-Ontańón A, Hamawandi A, Bertos-Quílez J (2022). Extramaxillary zygomatic implant coverage with a pedicled buccal fat pad flap through a tunnel approach: a prospective case series. Int J Oral Maxillofac Implants.

[80] Zwahlen RA, Grätz KW, Oechslin CK, Studer SP (2006). Survival rate of zygomatic implants in atrophic or partially resected maxillae prior to functional loading: a retrospective clinical report. Int J Oral Maxillofac Implants.

[81] Aparicio C, Manresa C, Francisco K, Claros P, Alández J, González-Martín O (2014). Zygomatic implants: indications, techniques and outcomes, and the zygomatic success code. Periodontol.

[82] Aparicio C, López-Píriz R, Peñarrocha M (2021). Preoperative evaluation and treatment planning. Zygomatic implant critical zone (ZICZ) location. Atlas Oral Maxillofac Surg Clin N Am.

[83] Alterman M, Fleissig Y, Casap N (2021). Zygomatic implants: placement considerations in implant-supported maxillary prosthesis. Atlas Oral Maxillofac Surg Clin N Am.

[84] Andre A, Dym H (2021). Zygomatic implants: a review of a treatment alternative for the severely atrophic maxilla. Atlas Oral Maxillofac Surg Clin N Am.

[85] Migliorança RM, Coppedê A, Dias Rezende RC, de Mayo T (2011). Restoration of the edentulous maxilla using extrasinus zygomatic implants combined with anterior conventional implants—a retrospective study. Int J Oral Maxillofac Implants.

[86] Coppedê A, de Mayo T, de Sá ZM, Amorin R, de Pádua APA, Shibli JA (2017). Three-year clinical prospective follow-up of extrasinus zygomatic implants for the rehabilitation of the atrophic maxilla. Clin Implant Dent Relat Res.

[87] Pellegrino G, Basile F, Relics D, Ferri A, Grande F, Tarsitano A (2020). Computer-aided rehabilitation supported by zygomatic implants: a cohort study comparing atrophic with oncologic patients after five years of follow-up. J Clin Med.

[88] Butterworth CJ, Lowe D, Rogers SN (2022). The zygomatic implant perforated (ZIP) flap reconstructive technique for the management of low-level maxillary malignancy—clinical & patient related outcomes on 35 consecutively treated patients. Head Neck.

[89] Cawood JI, Howell RA (1988). A classification of the edentulous jaws. Int J Oral Maxillofac Surg.

[90] Bedrossian E, Sullivan RM, Fortin Y, Malo P, Indresano T (2008). Fixed-prosthetic implant restoration of the edentulous maxilla: a systematic pretreatment evaluation method. J Oral Maxillofac Surg.

[91] Bedrossian E, Bedrossian EA (2019). Systematic treatment planning protocol of the edentulous maxilla for an implant-supported fixed prosthesis. Compend Contin Educ Dent.

[92] Bedrossian E, Bedrossian E, Bedrossian EA, Brecht LE (2022). Systematic treatment planning protocol for the maxilla. The immediacy concept.

[93] Aparicio C, Ouazzani W, Garcia R, Arevalo X, Muela R, Fortes V (2006). A prospective clinical study on titanium implants in the zygomatic arch for prosthetic rehabilitation of the atrophic edentulous maxilla with a follow-up of 6 months to 5 years. Clin Implant Dent Relat Res.

[94] Aparicio C, Ouazzani W, Aparicio A, Fortes V, Muela R, Pascual A (2010). Immediate/early loading of zygomatic implants: clinical experiences after 2 to 5 years of follow-up. Clin Implant Dent Relat Res.

[95] Aparicio C, Manresa C, Francisco K, Aparicio A, Nunes J, Claros P (2014). Zygomatic implants placed using the zygomatic anatomy-guided approach versus the classical technique: a proposed system to report rhinosinusitis diagnosis. Clin Implant Dent Relat Res.

[96] Esposito M, Barausse C, Balercia A, Pistilli R, Ippolito DR, Felice P (2017). Conventional drills vs piezoelectric surgery preparation for placement of four immediately loaded zygomatic oncology implants in edentulous maxillae- results from 1-year split-mouth randomised controlled trial. Eur J Oral Implantol.

[97] Balaji S, Balaji P (2020). Comparative evaluation of direct sinus lift with bone graft and zygoma implant for atrophic maxilla. Indian J Dent Res.

[98] Borgonovo A, Grandi T, Vassallo S, Signorini L (2021). Extrasinus zygomatic implants for the immediate rehabilitation of the atrophic maxilla: 1-year postloading results from a multicenter prospective cohort study. J Oral Maxillofac Surg.

[99] Tao B, Shen Y, Sun Y, Huang W, Wang F, Wu Y (2020). Comparative accuracy of cone-beam CT and conventional multislice computed tomography for real-time navigation in zygomatic implant surgery. Clin Implant Dent Relat Res.

[100] Malevez C, Abarca M, Durdu F, Daelemans P (2004). Clinical outcome of 103 consecutive zygomatic implants: a 6–48 months follow-up study. Clin Oral Implants Res.

[101] Davó R, Malevez C, Pons O (2013). Immediately loaded zygomatic implants: a 5-year prospective study. Eur J Oral Implantol.

[102] Yates JM, Brook IM, Patel RR, Wragg PF, Atkins SA, El-Awa A (2014). Treatment of the edentulous atrophic maxilla using zygomatic implants: evaluation of survival rates over 5–10 years. Int J Oral Maxillofac Surg.

[103] Ramezanzade S, Yates J, Tuminelli FJ, Keyhan SO, Yousefi P, Lopez-Lopez J (2021). Zygomatic implants placed in atrophic maxilla: an overview of current systematic reviews and meta-analysis. Maxillofac Plast Reconstr Surg.

[104] Neugarten J, Tuminelli F, Walter L (2017). Two bilateral zygomatic implants placed and immediately loaded: a retrospective chart review with up-to-54-month follow-up. Int J Oral Maxillofac Implants.

[105] Nkenke E, Hahn M, Lell M, Wiltfang J, Schultze-Mosgau S, Stech B (2003). Anatomic site evaluation of the zygomatic bone for dental implant placement. Clin Oral Implants Res.

[106] Araújo R, Sverzut A, Trivellato A, Sverzut C (2017). Retrospective analysis of 129 consecutive zygomatic implants used to rehabilitate severely resorbed maxillae in a two-stage protocol. Int J Oral Maxillofac Implants.

[107] Dawson A, Martin W, Polido W (2022). The SAC classification in implant dentistry. The SAC classification in implant dentistry.

[108] da Hora Sales PH, Gomes MVSW, de Oliveira-Neto OB, de Lima FJC, Leão JC (2020). Quality assessment of systematic reviews regarding the effectiveness of zygomatic implants: an overview of systematic reviews. Med Oral Patologia Oral y Cirugia Bucal.

[109] Davó R, David L (2019). Quad zygoma: technique and realities. Oral Maxillofac Surg Clin N Am.

[110] Butterworth CJ (2019). Primary vs secondary zygomatic implant placement in patients with head and neck cancer—a 10-year prospective study. Head Neck.

[111] Chana H, Smith G, Bansal H, Zahra D (2019). A retrospective cohort study of the survival rate of 88 zygomatic implants placed over an 18-year period. Int J Oral Maxillofac Implants.

